# The Accordion Maneuver: A Noninvasive Strategy for Absent or Delayed Callus Formation in Cases of Limb Lengthening

**DOI:** 10.1155/2015/912790

**Published:** 2015-10-19

**Authors:** Asim M. Makhdom, Adrian Sever Cartaleanu, Juan Sebastian Rendon, Isabelle Villemure, Reggie C. Hamdy

**Affiliations:** ^1^Division of Orthopaedic Surgery, Shriners Hospital for Children, Montreal Children Hospital, McGill University, 1529 Cedar Avenue, Montreal, QC, Canada H3G 1A6; ^2^Department of Orthopaedic Surgery, King Abdulaziz University, Jeddah 21589, Saudi Arabia; ^3^Canada Research Chair in Mechanobiology of the Pediatric Musculoskeletal System, Department of Mechanical Engineering, Polytechnique Montreal, Montreal, QC, Canada

## Abstract

The distraction osteogenesis (DO) technique has been used worldwide to treat many orthopaedic conditions. Although successful, absent or delayed callus formation in the distraction gap can lead to significant morbidities. An alternate cycle of distraction-compression (accordion maneuver) is one approach to accelerate bone regeneration. The primary aim of our study is to report our experience with the accordion maneuver during DO and to provide a detailed description of this technique, as performed in our center. The secondary aim is to present a review of the literature regarding the use of accordion maneuver. We reviewed the database of all patients undergoing limb lengthening from the year of 1997 to 2012. Four patients (6.15%) out of 65 showed poor bone regenerate in their tibiae and therefore accordion maneuver was applied for a mean of 6.75 weeks. Of these, three patients have had successful outcome with this technique. The literature showed that this technique is successful approach to trigger bone healing. However, details of how and when to apply this combination of distraction-compression forces were lacking. In conclusion, the accordion technique is safe noninvasive approach to promote bone formation, thus avoiding more invasive surgical procedures in cases of poor callus formation in limb lengthening.

## 1. Introduction

Distraction osteogenesis (DO) is a surgical technique used worldwide to treat a broad variety of musculoskeletal and craniofacial conditions, including correction of angular deformities or management of bone defects secondary to infection, trauma, or tumor via limb lengthening or segmental bone transport [1, 2]. This technique was popularized by Ilizarov in the early 1950s who demonstrated that when controlled gradual distraction is applied to the two ends of a bone following a low energy osteotomy, new bone will form in the distracted gap [2]. Its principle is based on the intrinsic capacity of the bone to regenerate under a controlled mechanical environment and is considered the best type of* in vivo* bone tissue engineering technique [3]. Both the rate and rhythm of distraction are vital to the quality of the regenerate bone.

Although DO is associated with satisfactory outcomes in most cases, absent or delayed callus formation in the distraction gap may occur. This could lead to significant morbidities, as the fixator needs to be kept in place for an extended period of time until the bone is completely consolidated. Consequently, unfavorable psychological impact, increased pin tract infections, persistent pain, and increased risk of osteopenia might be encountered [4–6]. In some cases, subsequent surgical interventions might be required [1, 4, 6]. Numerous techniques have been described in the management of poor regenerate in cases of DO, including systemic administration of pharmaceutical agents such as bisphosphonates, local exogenous administration of growth factors (GFs) such as BMPs, bone marrow cells (BMC), and the use of externally applied low-intensity pulsed ultrasound (LIPU) and pulsed electromagnetic fields (PEMF) [3, 7–10].

There are several modalities where the use of compressive forces in the context of DO could be used in order to accelerate bone formation in the distracted gap, and these include early and increasing weight bearing on the operated limb, dynamization of the fixator, overdistraction, and then shortening and alternating cycles of distraction and compression [11–13]. This last technique—the accordion maneuver—has originally been described by Ilizarov in order accelerate bone regeneration in DO [2]. However, despite several reports in the English literature on the successful use of this technique in the management of poor regenerate, they are mostly anecdotal without a detailed description of this maneuver [14–23].

The aim of this study is to report our experience with the accordion maneuver in a small series of cases with absent or delayed bone formation during DO and to provide a detailed description of this technique, as performed in our center. We also present a review of the literature regarding the use of alternating cycles of distraction and compression in cases of DO, nonunions, and fractures in both human and animal studies.

## 2. Patients and Methods

After approval from our local institutional review board, we retrospectively reviewed all patients who underwent straight lower limb lengthening at our institution between 1997 and 2012. The medical records of 65 patients (forty-one males and twenty-four females, M : F = 1.7 : 1) who underwent 72 interventions (35 on right side, 37 on left side), in which 72 bone segments were lengthened (44 femora and 28 tibiae), were reviewed. Of these 4 patients underwent the accordion technique. The demographic data, clinical course and imaging information, diagnosis, surgery, lengthening details, and complications were all collected from the medical record system. In all patients, a low energy osteotomy was performed by creating multiple small drill holes at the site of osteotomy followed by completion of the osteotomy with an osteotome. Immediate weight bearing as tolerated was initiated in all patients with intense physiotherapy.

The specific indication for using the accordion maneuver was an absent or delayed callus formation in the distraction gap, judged radiographically. The accordion maneuver consisted in alternating distraction with compression as follows: distraction (0.25 mm) in the morning and then compression (0.25 mm) in the afternoon, followed by distraction (0.25 mm) in the evening, resulting in an overall daily lengthening of 0.25 mm.

## 3. Results

**Figure 1:**
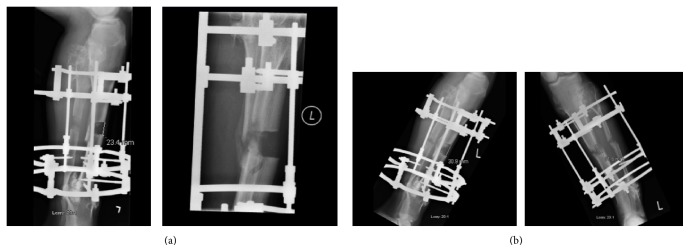
(a) Anteroposterior (on the right) and lateral radiographic (on the left) views of left tibia (case 4) showing very discrete bone regenerate after 6 weeks of distraction at a rate of 0.25 mm × 2 times/day. (b) Anteroposterior (on the right) and lateral radiographic (on the left) views of left tibia (case 4) after 7 weeks of the accordion technique showing significantly improved osteoformation.

The decision to apply the accordion maneuver was taken during initial distraction when imaging has shown absent or significantly delayed callus formation in the distraction gap. This was the case of tibiae in four patients (6.15%) of the 65 investigated. Their mean age was 16.5 years (range, 10 to 20 years). After lengthening initiation, their X-rays showed an absent or very timid bone regenerate in the distraction gap (Figure 1(a)). In these four cases, the accordion maneuver was applied at a mean of 4.5 weeks after surgery (range, 3–7 weeks), which corresponds to a mean of 3.62 weeks (range, 2–6 weeks) after initiation of the distraction phase. The accordion maneuver was carried out on a daily basis, alternating distraction with compression three times per day, for an average of 6.75 weeks, as previously described. The total distraction period (the routine distraction period + the accordion maneuver period) was of an average of 12.5 weeks (range, 11–14 weeks) to obtain a mean lengthening of 3.92 cm (range 3–5 cm). The residual limb length discrepancy was on average 1.12 cm (range, 0.7–2 cm). A mean healing index of 75.38 days/cm was noted. Details on clinical and accordion maneuver details are provided in Table 1.

**Figure 2:**
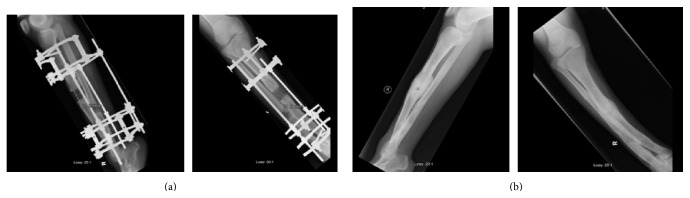
(a) Anteroposterior (on the right) and lateral radiographic (on the left) views of left tibia (case 3) showing insufficient response after 4 weeks of accordion maneuver. (b) Anteroposterior (on the right) and lateral radiographic view (on the left) of left tibia (case 3) showing complete healing after bone grafting and BMP-7 administration.

Favorable progression of the bone regenerate was noted after an average of 5.3 weeks (range, 4–6 weeks) after starting the accordion maneuver in three out of four patients (Figure 1(b)). These patients continued to have full bone consolidation in the distraction gap. However, in one patient (case number 3), infection has complicated the course and there was absent bone formation after using the accordion maneuver (Figure 2(a)). Antibiotic treatment, additional bone grafting, and administration of bone morphogenetic protein-7 (OP-1) ultimately resulted in bone union for this patient (Figure 2(b)).

## 4. Discussion

Several host related, local, and iatrogenic causes can lead to poor bone regenerate during DO [8]. These include systemic illness, infection, immunosuppression, poor tissue envelope, exposure to radiation, instability of the external fixator, suboptimal osteotomy technique, and rapid distraction rate [8]. We were unable to identify any of these risk factors in 3 out of the 4 patients with poor regenerate and the application of the accordion maneuver in these 3 patients resulted in successful bone regeneration in the distracted gap, while, in the fourth patient (case number 3), the accordion maneuver failed to stimulate the regenerative process. We believe this is most likely due to the presence of underlying infection. This emphasizes the importance of identifying all risk factors that may lead to a poor regenerate in DO before the use of the accordion technique. However, a firm conclusion can be made when a larger sample size is studied.

A review of the English literature revealed several clinical studies in humans reporting the use of the accordion technique in cases with poor regenerate bone formation in DO, the majority of them with positive outcome. However, the description of the alternate distraction-compression regimen in these studies is anecdotal and lacks details as of when, how, and for how long this technique is applied (Table 2) [15–17, 19, 24–28].

The accordion maneuver has also been used clinically to stimulate bone formation in the context of fracture healing. Similar to its reported use in DO, most of these studies also reported positive outcome, however still with poor description of the technique (Table 3) [14, 18, 29–31]. Interestingly, only experimental studies performed in animals have provided details of this technique. Mofid et al. showed that daily sequential compression and distraction for 3 weeks during the consolidation phase at rate of 1 mm/day increased significantly the bone formation when compared to the control group in mandibular DO in rabbit model [21]. Claes et al. investigated the effect of temporary distraction and compression on bone regeneration in fracture healing [11]. The authors noted higher bone formation in the treatment group when compared with the control group. On the other hand, Greenwald et al. used a rat mandibular DO model and reported that there were no differences histologically and radiographically between groups of rats with distraction-compression protocol versus a control group with standard DO technique [23]. We could not explain why these negative results were obtained, except that the regimen used by these authors was not an accordion technique with alternating cycles of distraction and compression, but rather 5 days of distraction followed by 2 days of compression. However, taken together, both clinical and experimental studies demonstrated the positive role of accordion maneuver in acceleration of bone regeneration in the context of both fracture healing and DO. However, the rate and rhythm of the accordion technique varied between experimental studies and were not available in the clinical studies; therefore, it is difficult to conclude which accordion regimen gives the best results.

**Figure 3:**
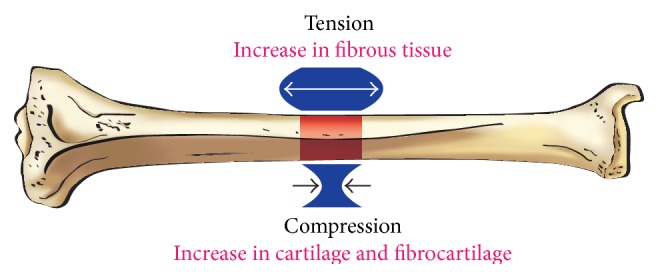
Illustration showing the osteogenic histological outcomes of tension versus compression.

From a mechanistic approach, it would be interesting to understand why the addition of compressive forces to those of distraction in cases of DO (the accordion technique) may lead to successful stimulation of bone formation in the distracted gap. It is well known that the mechanical environment plays a major role in bone formation (osteogenesis and chondrogenesis) and that bones adapt to the mechanical loads they are subjected to in terms of modeling, remodeling, and regeneration (Wolff's law) [32]. Interestingly, experimental studies showed that dynamic compression has greater bone remodeling than static compression [33]. One explanation is that the skeleton requires “time off” from mechanical loading as bone cells desensitize promptly from the mechanical stimulation, and resensitization must happen before the cell can transduce any prospective mechanical loads into biochemical signals [34]. In DO, mechanical loads can take the form of compressive, tensile (distraction), or shear forces. Not all these forces have equal effect on bone formation. It has been demonstrated that the application of various types of loads may have different effects on the differentiation of mesenchymal stem cells and may ultimately decide the fate of progenitor cells exposed to these loads: osteogenic versus chondrogenic fate. Compressive forces may lead to fibrogenesis, osteogenesis, and intramembranous bone formation, while distraction forces may lead to chondrogenesis and endochondral bone formation (Figure 3) [35].

In the context of standard technique of DO, most of the forces generated during the lengthening process are believed to be tensile forces. The addition of outside compressive forces during the lengthening process has been reported to be beneficial for bone formation, whether in the form of weight bearing, compression after overdistraction, or dynamization of the fixator or as mentioned by the accordion maneuver [2]. All these have been shown to be beneficial for regenerate bone formation in the distracted gap. In the only study that we were able to find, directly comparing the effects of compression versus distraction, Hente et al. observed that the amount of periosteal callus formation was up to 25 times greater on the compression side when compared to the distraction side in an experimental model of tibial fractures, using a specially designed external fixator [36]. This may explain the positive effect of adding “compression” during the accordion maneuver.

At the molecular level, numerous studies have analyzed the expression of various cytokines, growth factors, and other molecules in the context of DO [3, 37–39]. However, to the best of our knowledge, no study directly analyzed the molecular changes as a result of application of the accordion technique and compared these changes to standard distraction protocols without compression. Thus, at the molecular level, the mechanism of action of the accordion technique remains largely unknown.

All the above-mentioned studies lead us to believe that the addition of compressive forces in the context of DO could have a positive effect in the stimulation of regenerate bone in the distracted gap. However, how frequent these compressive forces should be applied in order to provide optimal results, for how long, and when during the lengthening process remain unanswered questions.

Our report has some limitations. This is very small retrospective case series with an absence of a control group. However, delayed or absent bone formation is a rare complication during limb lengthening and it will be difficult to study a large cohort of patients with such complication from a single institution. Additionally, although it appears from the literature review that the effect of compression has a major role for bone formation, we were not able to determine whether the slow speed in the distraction rate or the effect of compression has contributed to successful bone formation in our patients. This can be determined by experimental laboratory studies and/or multi-institutional clinical investigations.

In conclusion, we believe that, in our small series, the accordion regimen described in this study may be successful in triggering the osteogenic potential of a poor regenerate, thus avoiding more invasive surgical procedures. The literature showed that the accordion maneuver is a successful approach to trigger bone healing. However, details of how and when to apply this combination of distraction-compression forces were lacking. Further research in the form of multi-institutional clinical as well as experimental studies is needed in order to optimize the use of the accordion technique as a noninvasive and nonpharmaceutical method to stimulate bone formation, not only in the context of DO but also in other bony pathologies with poor bone formation. Finally, our future understanding of mechanotransduction in DO might extend the indications of the accordion maneuver to be used not only in cases of poor regenerate, but also during standard lengthening procedures to accelerate bone regeneration.

## Figures and Tables

**Table 1:** Clinical data for patients who underwent the accordion maneuver.

Data	Case number 1	Case number 2	Case number 3	Case number 4
Age and gender	18 y.o., female	10 y.o., male	20 y.o., male	18 y.o., male

Diagnosis	Blount disease	Fibular hemimelia	Tibial hemimelia	HME^1^

Indication for surgery, LLD	LLD, L < R 6 cm	LLD, L < R 5.2 cm	LLD, R < L 5 cm	LLD, L < R 4 cm

Surgical procedure	Tibial lengthening with circular Ilizarov	Tibial lengthening with circular Ilizarov	Tibial lengthening with circular Ilizarov	Tibial lengthening with circular Ilizarov

Comorbidities	None	None	None	None

Start time of accordion maneuver	5 weeks after surgery	4 weeks after surgery	3 weeks after surgery	7 weeks after surgery

Lengthening process description	Latency period: 4 days Distraction: 0.25 mm × 4 times (1 mm)/day for 4.5 weeks and then the accordion maneuver^2^ was initiated and lasted for 7 weeks Final distraction: 0.25 mm × 2 times (0.5 mm)/day for 2 weeks	Latency period: 6 days Initial distraction: 0.25 mm × 2 times (0.5 mm)/day for 2 weeks and then the accordion maneuver was initiated and lasted for 4 weeks Final distraction: 0.25 mm × 4 times (1 mm)/day for 2 weeks and then 0.25 mm × 2 times (0.5 mm)/day for 4 weeks	Latency period: 8 days Initial distraction: 0.25 mm distraction × 3 times (0.75 mm)/day for 2 weeks, and then the accordion maneuver was initiated and lasted for 9 weeks, stopping the lengthening thereafter	Latency period: 6 days Initial distraction: 0.25 mm distraction × 2 times (0.5 mm)/day for 6 weeks, and then the accordion maneuver was initiated and lasted for 7 weeks, stopping the lengthening thereafter

Total lengthening duration	14 weeks	12 weeks	11 weeks	13 weeks

Lengthening achieved	5 cm	4.4 cm	3 cm	3.3 cm

Residual LLD^3^	1 cm	0.8 cm	2 cm	0.7 cm

Lengthening index	19.6 days/cm	19.09 days/cm	25.66 days/cm	27.57 days/cm

Healing index	52.8 days/cm	54.1 days/cm	122.5 days/cm	72.12 days/cm

Outcome and complications	Bone regenerate observed at 6 weeks within the accordion. Had transient peroneal nerve palsy	Bone regenerate observed at 4 weeks within the accordion. Had 5 degrees of knee flexion contracture. 3 weeks after frame removal (9-month post-op), the regenerate was fractured and was nailed, ultimately healed	No bone regenerate formed after the accordion maneuver. Had infection, was successfully treated with antibiotics, and was followed up 7 months later by bone grafting and OP-1^4^. Residual bowing of the tibia. 40 degrees fixed equinus R ankle	Good bone regenerate observed at 6 weeks within the accordion course with no complications

Comments	Underwent concomitant correction of valgus	After 4 weeks of accordion, continued with 1 mm distraction/day for 2 weeks, had fibular premature fusion, and underwent reosteotomy and then continued distraction for another 4 weeks	Infection treated with antibiotics. Absent bone formation after using the accordion maneuver	Had correction of valgus. After finishing the accordion, started distraction 1 mm/day, for 2 days had pain, and stopped

^1^HME: Hereditary Multiple Exostoses.

^2^Accordion maneuver: 0.25 mm distraction in AM, followed by 0.25 mm compression early PM, and then distraction of 0.25 mm late PM (0.25 mm of lengthening/day).

^3^LLD: limb length discrepancy.

^4^OP-1: osteogenic protein-1.

**Table 2:** Previously published clinical studies reporting the accordion technique during delayed or absent callus formation of distraction osteogenesis (DO).

Authors	Number of patients	Indication	Successful outcome	Technique for accordion maneuver
Iacobellis et al. 2010 [26]	3	Poor regenerate during bone transport	100% (3/3)	Compression followed by distraction of the transport segment (no details)

Hatzokos et al. 2011 [24]	8	Delayed consolidation	75% (6/8)	Accordion technique (no details).

Kawoosa et al. 2003 [25]	1	Delayed consolidation	100% (1/1)	Alternate compression and distraction of the regenerate (no details)

El-Mowafi et al. 2005 [27]	*N* = ?	Delayed consolidation	?	Compression and distraction of a moving segment (no details)

El-Sayed et al. 2010 [17]	25	Absence of callus formation	76% (19/25)	Distraction-compression technique (no details)

Tsuchiya et al. 1997 [28]	*N* = ?	Poor regenerate during bone transport	?	Compression and distraction of a moving segment (no details)

Vidyadhara and Rao 2007 [15]	*N* = ?	Poor regenerate callus during bone transport	?	Compression and distraction of a moving segment (no details).

Simpson and Kenwright 2000 [16]	2	Poor callus formation	0% (0/2)	Changes in the dynamics of distraction (no details)

Krishnan et al. 2006 [19]	2	Poor regenerate during bone transport	100% (2/2)	Reported as distraction, discontinued, reversed, and restarted at a reduced rate (0.25 mm/12 h, instead of 0.25 mm/6 h)

**Table 3:** Previous reports on the use of distraction and compression in treatment of long bone fractures, delayed unions, and nonunions.

Authors	Number of patients	Indication	Successful outcome	Technique for distraction-compression
Kulkarni 2004 [29]	N/A	Hypertrophic nonunion	N/A	Distraction 0.5 mm/day for 20 days, then stopping for the next 20 days, and finally compression

Inan et al. 2005 [30]	11	Femoral pseudarthrosis	100% (11/11)	Cyclic compression and distraction at the nonunion site

Madhusudhan et al. 2008 [14]	2	Tibial nonunion	100% (2/2)	Compression and distraction (no details)

Laursen et al. 2000 [18]	2	Tibial nonunions	50% (1/2)	Alternating distraction (1 week) with compression (1 week), until callus visible on X-ray

Chand et al. 2010 [31]	2	Nonunion of long bone fractures	100% (2/2)	Compression and distraction technique (no details)
